# A macroevolutionary perspective of cryptic coloration in sexually dichromatic grasshoppers of the genus *Sphenarium* (Orthoptera: Pyrgomorphidae)

**DOI:** 10.1007/s00442-024-05643-7

**Published:** 2025-01-10

**Authors:** Víctor Hugo Ramírez-Delgado, Martín Alejandro Serrano-Meneses, Raúl Cueva del Castillo

**Affiliations:** 1 Laboratorio de Ecología, UBIPRO, FES Iztacala, Universidad Nacional Autónoma de México, Tlalnepantla, 54090 México; 2 Departamento de Ecología Tropical, Campus de Ciencias Biológicas y Agropecuarias, Universidad Autónoma de Yucatán, Itzimná, 97000 Mérida, Yucatán México; 3 Departamento de Ciencias Químico Biológicas, Universidad de Las Américas Puebla, San Andrés Cholula, 72810 Puebla, México

**Keywords:** Background matching, Comparative analysis, Crypsis, Disruptive coloration, Grasshoppers, Sexual dichromatism

## Abstract

Background matching and disruptive coloration are defense mechanisms of animals against visual predators. Disruptive coloration tends to evolve in microhabitats that are visually heterogeneous, while background matching is favored in microhabitats that are chromatically homogeneous. Controlling for the phylogeny, we explored the evolution of the coloration and the marking patterns in the sexual dichromatic and widely distributed neotropical grasshoppers of the genus *Sphenarium*. These grasshoppers represent an excellent model to investigate the evolution of cryptic coloration on insects due to the heterogeneity of the environments where they have evolved. We found a correlation between the grasshoppers' coloration and disruptive markings with the chromatic properties of their environments that was inferred by the levels of precipitation during the rainy season. The results suggest that colors and marking patterns could evolve due to predation pressures. Color in both sexes could offer camouflage that is not perfectly background matched to a single habitat but instead offers a degree of resemblance to multiple backgrounds. Moreover, we found that males and females chromatic properties differ between them and precipitation levels where the species are found. This suggests that the sexes have diverged in their response to the environments, favoring the evolution of sexual dichromatism in these grasshoppers.

**Supplementary Information:**

The online version contains supplementary material available at 10.1007/s00442-024-05643-7.

## Introduction

In many animal taxa, coloration can be adaptive in several ways, including social signaling, thermoregulation, protection from ultraviolet light, and antipredator defenses (Cott 1940; Cuthill et al. 2017). Cryptic coloration is adaptive because it reduces the signal produced by an organism; it thus becomes less visually conspicuous to potential predators (Bond 2007; Théry and Gomez 2010). Two of the most widespread strategies to reach cryptic coloration are background matching (BM) and disruptive coloration (DC) (Stevens et al. 2006a; Stevens and Merilaita 2009; Quicke 2017; de Alcantara Viana et al. 2022). BM is the resemblance between the colors and patterns of an organism with its surroundings (Hughes et al. 2019). DC is the concealment of an organism’s outline due to contrasting markings that break or distract the attention of the predators from the organism's outline (Stevens and Cuthill 2006; Stevens and Merilaita 2009).

BM can be favored in chromatically homogeneous habitats (Robledo-Ospina et al. 2017; Orton and McBrayer 2019); its success depends on the coloration of the environment and the probability that an individual stays on the backgrounds where it is cryptic (Merilaita et al. 1999; Kang et al. 2016). However, BM can be ineffective at reducing the risk of detection when animals are in motion in heterogeneous environments (Ioannou and Krause 2009). DC can co-occur with BM and enhances concealment from various predators (Cuthill et al. 2005; Schaefer and Stobbe 2006; Fraser et al. 2007; Adams et al. 2019). DC success could be maximized when some marks on the organism’s surface match the background and other marks have high contrast with the rest of the body (Stevens and Merilaita 2009). DC also has evolved in visually heterogeneous microhabitats because it breaks the outlines of the organisms independently of the variable background patterns and can conceal an organism while it is in motion (Stevens et al. 2006a, b).

The evolution of BM colors represents a compromise between matching closely a single background or adopting a generalist strategy where the individuals resemble multiple backgrounds to some extent but not perfectly (Hughes et al. 2019). On the other hand, DC could be independent of the background coloration and depends on the organisms’ markings and shape (Merilaita and Lind 2005). DC could be more adaptive than BM in individuals with high vagility, which have a low probability of staying in specific backgrounds or inhabiting heterogeneous color habitats (Théry 2007; Théry and Gomez 2010; Robledo-Ospina et al. 2017).

Because BM and DC strategies decrease the probability of detection by predators, selection will act on organisms so that they match the geographical variation in substrate color (Endler 1990; Stuart-Fox and Ord 2004; Rosenblum 2006; Marshall et al. 2015; Hantak and Kuchta 2018; Adams 2019). For instance, in some arthropod taxa, BM and DC tactics have evolved closely with the environment. Jumping spiders exhibit BM when in relatively flat chromatic backgrounds, whereas in dynamically changing backgrounds, they exhibit DC (Robledo-Ospina et al. 2017). Also, crabs living in homogeneous backgrounds evolved BM, whereas those living in heterogeneous backgrounds have evolved DC (Price et al. 2019).

In many grasshopper taxa, cryptic coloration has evolved as an anti-predator strategy (Tsurui et al. 2010, 2013; Edelaar et al. 2019; Camacho et al. 2020); even different morphs of a given grasshopper species have been described as disruptive or showing resemblance to their environment (Tsurui et al. 2013; Forsman 2018; Ramírez-Delgado and Cueva del Castillo 2020). However, if males and females occupy different microhabitats because of their different sexual roles, natural selection could favor a divergence between females and males (Slatkin 1984), thereby favoring sex-specific cryptic color patterns (Forsman and Appelqvist 1999; Medina et al. 2016; Ramírez-Delgado and Cueva del Castillo 2020; Cueva del Castillo et al. 2021).

The evolution of sexual dichromatism has been extensively studied in the contexts of both sexual (Andersson 1994) and natural selection (Font et al. 2009; Orton and McBrayer 2019). Although most research has emphasized sexual selection, there is growing evidence that natural selection also plays a critical role. This has been documented across a wide range of taxa, including both vertebrates (Andersson 1994) and invertebrates (Wiens and Tuschhoff 2020). Insects such as Lepidoptera, Diptera, and Odonata have been shown to exhibit sexual dichromatism driven by both inter- and intrasexual selection (Wiernasz 1989; Grether1996; Lederhouse et al. 1996; Katayama et al. 2014; Quicke 2017, and references therein). Furthermore, differential selective pressures from visual predators have been implicated in the evolution of sexual dichromatism in vertebrates (Font et al. 2009; Orton and McBrayer 2019). However, in invertebrates, the relationship between sexual dichromatism and cryptic coloration remains relatively understudied (Forsman and Appelqvist 1999; Ramírez-Delgado and Cueva del Castillo 2020, 2023; Cueva del Castillo et al. 2021).

Grasshoppers of the genus *Sphenarium* are found in a wide variety of environments and show high variation in their color and marking patterns (Sanabria-Urbán et al. 2017). Moreover, some species of this genus exhibit a notorious sexual dichromatism; BM is associated with females and DC to males (Ramírez-Delgado and Cueva del Castillo 2020, 2023; Cueva del Castillo et al. 2021). In *S. zapotecum*, in visually heterogeneous areas, predators spent more time searching for striped male morphs with lower BM and higher disruptive properties. On the other hand, females with high BM improve survival and significantly increase predator searching time (Ramírez-Delgado and Cueva del Castillo 2023). *Sphenarium* grasshoppers are the prey of many visually oriented vertebrates, including birds, mammals, reptiles (Kevan 1977), and humans, which have extensively consumed species of the genus since pre-Columbian times (Ortiz de Montellano 1978; Ramos-Elorduy and Pino-Moreno 1989).

The *Sphenarium* genus consists of 17 flightless univoltine generalist herbivorous species distributed from central Mexico to northern Guatemala in a high altitudinal margin (0 to > 2600 m above sea level, Sanabria-Urbán et al. 2015). Because the differences in altitude and climate associated with their distribution are substantial, these species are found in rainy and dry deciduous tropical forests, temperate forests, shrublands, and grasslands. In low seasonal environments, the vegetation remains mainly greenish all year round, while in highly seasonal environments, there is a transition between brownish and greenish tonalities. The grasshoppers hatch in May and June during the rainy season and die during the coldest months (December to February) (Sanabria-Urbán et al. 2017). Females are larger than males (Sanabria-Urbán et al. 2017), and both sexes are polygamous (Cueva del Castillo and Núñez-Farfán 1999, 2002). The males tend to search actively for females, whereas females are less mobile and can be found close to the ground when they are about to lay their eggs (Ramírez-Delgado and Cueva del Castillo 2020).

The diversity of environments these grasshoppers inhabit allows us to analyze the potential adaptive chromatic cryptic divergences between them. Despite the implication of DC and BM on the evolution of the chromatic patterns of the species, very few comparative studies have tested their relative impact on the chromatic evolution of lineages (Caro and Koneru 2021). Moreover, given that many cryptic species are sexually dichromatic, focusing on these taxa may reveal profound implications regarding the evolution of intraspecific chromatic variation with respect to selective pressures imposed by visual predators on the members of each sex.

In this study, we explored the relationship between the coloration patterns of males and females of the grasshoppers of the genus *Sphenarium* and their environment marking patterns whilst controlling for phylogenetic effects. Because matching coloration between habitats and individuals can be impacted by seasonal and geographic climatic variation (Caro et al. 2016), we investigated the relationship between the environment and the grasshoppers' chromatic patterns by using the precipitation patterns in the localities and months where the adult grasshoppers were located. Since vegetation greenness is strongly related to rainfall and soil moisture (Davenport and Nicholson 1993; Huber et al. 2011; Tripathi et al. 2024), different grasshoppers’ colors and patterns are expected from different levels of greenness and projected shadows of the vegetation (Yom-Tov and Geffen 2006). Moreover, due to local adaptation and BM, we expected to find a positive relationship between environmental coloration and the overall coloration of males and females. We also hypothesized that this relationship would be stronger in females than in males due to their lower mobility (Ramírez-Delgado and Cueva del Castillo 2020; Cueva del Castillo et al. 2021). Additionally, since the water content in the diet of grasshoppers can affect their coloration (Otte and Williams 1972; Lymbery 1992; Umbers et al. 2014), we predicted a positive correlation between grasshopper coloration and environmental precipitation during both the wettest (PTW) and the driest (PDT) periods (see Sect. 2.3, Climatic data acquisition).

## Materials and methods

### Image acquisition

**Fig. 1:**
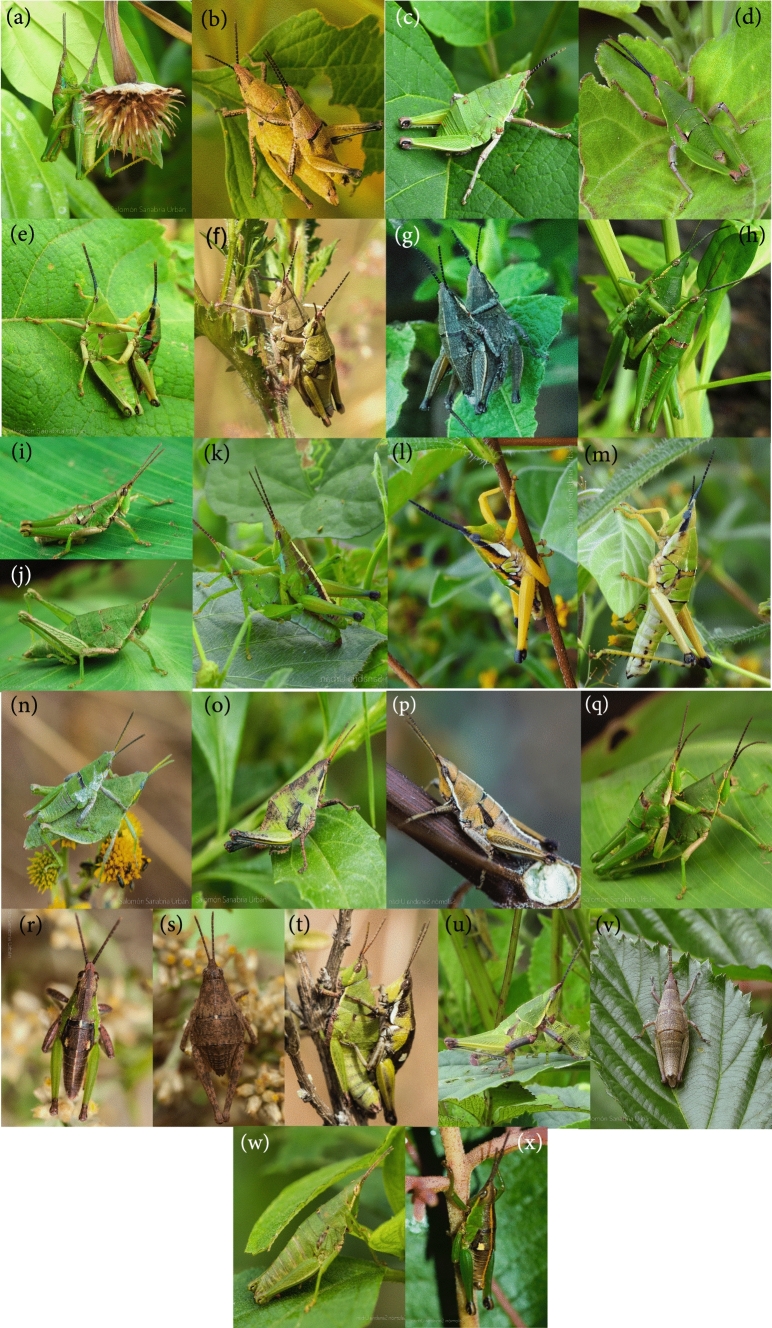
**a, b** Males and females of *Sphenarium* taxa. **a** Male (top) and a female (bottom) of *S. adelinidae*; **b** male and female *S. borrei*; **c** male and **d** female *S. crypticum*; **e** male and female *S. histrio*; **f** male and female *S. infernalis*; **g** male and female *S. macrophallicum*; **h** male and female *S. mexicanum*; male **i** and female **j** *S. minimum*; **k** male and female *S. miztecum*; male **l** and female **m** *S. occidentalis*; **n** male and female of *S. planum*; male **o** and female **p** (of *S. purpurascens*; **q** male and female of *S. totonacum*; male **r** and female **s** of *S. tarascum*; **t** male and female of *S. variabile*; male **u** and female **v** of *S. rugosum*; and male **w** and female **x** of *S. zapotecum*

Between October 2017 and September 2018, we obtained photographs of male and female adults of the 17 *Sphenarium taxa* (Fig. 1), as well as their backgrounds in 17 localities across central and southern Mexico (Fig. S1). The places varied in elevation from 77 to 2374 m above sea level (Table S1). Details of number of photographs taken per species, sex, and data obtained for color and pattern analyses are provided in supplementary material (Table S2). During data collection, the geographic position and elevation of each locality was recorded using a GPS-map 60CSx (Garmin, Kansas City, USA). In each location, we walked at a slow and steady pace whilst looking for grasshoppers in areas of approximately 100 m^2^; we registered the exact point where grasshoppers were found, and we caught them by hand. Once collected, the grasshoppers were placed in a cooler for approximately 5 min; this allowed us to lower the temperature and activity of individuals. We then placed each grasshopper back in the site where it was collected, we took a photograph of it against the background where it was found. We took the photographs below a white diffuser umbrella on sunny days. In the photographs, we included a color checker card (X-Rite Color Checker Passport 2, Munsell Color Laboratories), which also included a ruler and a grey color scale. The photographs were taken with a Canon EOS 70D digital camera, fitted with 18–55 mm, f/3.5–5.6 lens. The settings of the camera, lens, and illumination were constant in all photographs. The aperture of the sensor was set to f-stops f/5.6; the values of light sensitivity (ISO) were set to 400; the focal distance was set at 55 mm; the camera was held at approximately 40 to 50 cm apart from the objective. The only setting that was adjusted between photographs was shutter speed; in this way we prevented overexposure in photographs. After the photographs were taken, the grasshoppers were temporarily placed in a plastic bag to avoid their potential recapture, and they were released after all the caught individuals in each locality were photographed. We stored the images as RAW format. The camera has no modifications to allow ultraviolet sensitivity, so the photographs only contain information of the visible light spectrum. We believe this is not an issue, since measurements in the grasshopper *Tetrix japonica,* suggests very little ultraviolet light reflectance (Tsurui et al. 2010). To obtain the photographs and make them objectively measurable we followed the suggestions outlined by Stevens et al. (2007), and Troscianko and Stevens (2015).

### Image analysis

To analyze the images, we used the Multispectral Image Calibration and Analysis (MICA) plugin (Troscianko and Stevens 2015) available for ImageJ software (Schneider et al. 2012). MICA plugin uses linear data from the raw images, controls the light conditions variation with the gray scales from the color checker and creates a multispectral image made of a stack of the images corresponding to long wave (R Channel), medium wave (G channel), and short wave (B channel). The multispectral image made it possible take objective measurements from different channel reflectances, to later compare the color and pattern between our photographs. From the multispectral images, we measured the reflectance of the RGB channels of the grasshoppers’ dorsal surface, and in the same photo, a similar area adjacent to the grasshoppers was considered the background surface of the grasshoppers. We also performed a granularity analysis on both surfaces (Chiao et al. 2009; Stoddard and Stevens 2010).

#### Color analysis

We estimated brightness, saturation and hue from the RGB reflectance data. The three parameters allowed us to separate the achromatic (brightness) and chromatic (saturation and hue) properties of the images. Brightness refers to intensity of light on the image. Hue is determined by the dominant wavelength of the visible spectrum. It is the attribute that permits colors to be classified as red, yellow, green, blue, or an intermediate color, and saturation pertains the amount of white light mixed with a hue. High-saturation colors contain little or no white light.

Brightness was obtained using the means of the three channel values: (R + G + B) / 3. Saturation was calculated as the Euclidean distance between completely white, and the RGB values obtained in our photographs. By following this approach, large distance values represent high saturation. We obtained two hue values: Hue1 = R / G, and Hue2 = (R + G) / B. Hue1 high values denote more red reflectance, while low values represent more green reflectance. Hue2 high values represent more yellow reflectance, while low values represent blue reflectance. These were calculated this way following the principle of opponent channels, which is based in the way opponent color channels work to detect color (Osorio and Vorobyev 2005).

We must emphasize that the analyses are interpreted from a human visible spectrum perspective. Spectral sensitivity can be different in other possible predators such as birds or mice, and their prey detectability could involve elements that we did not consider in this study (Théry and Gomez 2010). Nonetheless, humans’ processing capabilities are similar to those of natural predators, especially birds (Dukas and Kamil 2001; de Alcantara Viana et al. 2022), and some studies have noticed that human “predation” can predict predation by other visual predators under natural conditions (Karpestam et al. 2013). Moreover, in central and southern Mexico, *S. zapotecum* and other species of the genus have been traditional elements of the human diet since pre-Columbian times (Ortiz de Montellano 1978; Ramos-Elorduy and Pino-Moreno 1989).

#### Pattern analysis

We performed a granularity (energy) analysis to measure the patterns in our photographs. This method roughly resembles the way animals decompose the visual information in different spatial frequencies (Stevens 2011). The photographs had to meet the following minimum pixel scale for this analysis 1 mm: 15 pixels; if the photographs did not meet this requirement, they were not used for pattern analysis (Troscianko and Stevens 2015). We used the average pixel reflectance of red and green channels to calculate the energy spectrum of grasshoppers and their background across 15 filters ranging from 2 to 256 pixels, in increments of multiples of √2. We obtained three descriptive values from this process: (i) overall pattern contrast: the amount of energy across all scales; (ii) the dominant marking size: the scale of dominant marking contrast (the filter where the highest value of energy is reached); and (iii) pattern diversity: the proportion between dominant marking contrast and rest of the measured energy.

### Climatic data acquisition

Because the *Sphenarium* species complete their reproductive cycle during the wettest and the driest periods of the year (Sanabria-Urbán et al. 2015), we obtained values from high-resolution climate surfaces (Fick and Hijmans 2017) of the precipitation of the wettest (PTW) and the driest (PDT) trimesters of the year for each sampling location. These parameters can be associated with the greenish levels of the localities at different times of the year. We expected green tonalities to be positively related with the amount of humidity and the amount of primary productivity of the plants, and brownish tonalities stronger associated with drier environments (Yom-Tov and Geffen 2006).

### Comparative analyses

Because lineages do not evolve independently from each other, and closely related taxa can share many traits (Harvey and Pagel 1991), it is necessary to take account of the phylogeny to explore the impact of natural selection in the diversification of lineages. To correct for the phylogenetic non-independence of taxa, we used the phylogenetic relationships of the *Sphenarium* genus based on mitochondrial and nuclear DNA sequences (Sanabria-Urbán et al. 2017), and the phylogenetic generalized least squares (PGLS) method to test the association between (a) the color and patterns of the grasshoppers, with (b) the colors and patterns of their environment and the climatic factors of the localities where the grasshoppers were found. PGLS were performed using the *caper* package (Orme et al. 2023) as implemented in R (ver. 4.0.1) (R Core Team 2020). PGLS is a comparative method that incorporates the phylogenetic autocorrelation of the data in the structure of errors (variance–covariance matrix; (Martins and Hansen 1997; Freckleton et al. 2002). In this case, the PGLS method was used to test the maximum likelihood of the evolutionary regression coefficient between traits (Pagel 1999). We also estimated the weighting parameter *λ* to improve the data's fit to the model and correct for phylogenetic effects in all generated PGLS models (Pagel 1999). *λ* measures phylogenetic dependence of observed trait data (Pagel 1999; Freckleton et al. 2002): the unit value approaches one when related species resemble each other more than they resemble species drawn at random from a phylogenetic tree (Blomberg and Garland Jr 2002).

### Testing the association between the coloration of grasshoppers, their background coloration, and climatic factors

We constructed four PGLS models for each sex to test for associations between the coloration parameters and climatic factors and background coloration. The natural log-transformed values of hue, saturation, and brightness of the grasshoppers were used as response variables, and the natural log-transformed values of hue, saturation, and brightness of the background, were used as respective explanatory variables. We further included the precipitation variables; ln(PWT) and ln(PDT) as explanatory variables in all models. Due to the multiple tests of association between variables, we adjusted the P values from the PLGS through a false discovery rate test using the Benjamini–Hochberg method (Benjamini and Hochberg 1995).

### Sexual dimorphism in color patterns

To test the divergence between the chromatic patterns of males and females of *Sphenarium* taxa, we performed major axis (MA) regressions of the independent contrasts of chromatic patterns of.

males on the independent contrasts of chromatic patterns of females. A slope steeper than 1 would mean that the divergence in the chromatic could be higher in males than females (> 1), whereas a slope lower than 1(< 1) would mean higher in females than males. We used the phylogenetic independent contrasts method (Felsenstein 1985), as implemented by the R package *caper* (Orme et al. 2023) to control for the phylogenetic non-independence of species (Harvey et al. 1991). We examined the studentized residuals looking for outliers >|± 3|, which were discarded from the analysis. Ultimately, we regressed the independent contrasts of the log-transformed values of hue1 and 2, saturation, brightness, overall patterns’ contrast, dominant marking size, and pattern diversity of the male grasshoppers on their counterparts on females by fitting major axis regression using the R package *smatr* (Warton et al. 2012). We selected MA regression because this method is appropriate when the purpose of line-fitting is not to predict Y from X, but to summarize the relationship between two variables, describing how traits are related through a linear relationship (Warton et al. 2016).

## Results

### *Association between grasshoppers’ coloration and background coloration* + *climatic factors*

**Fig. 2:**
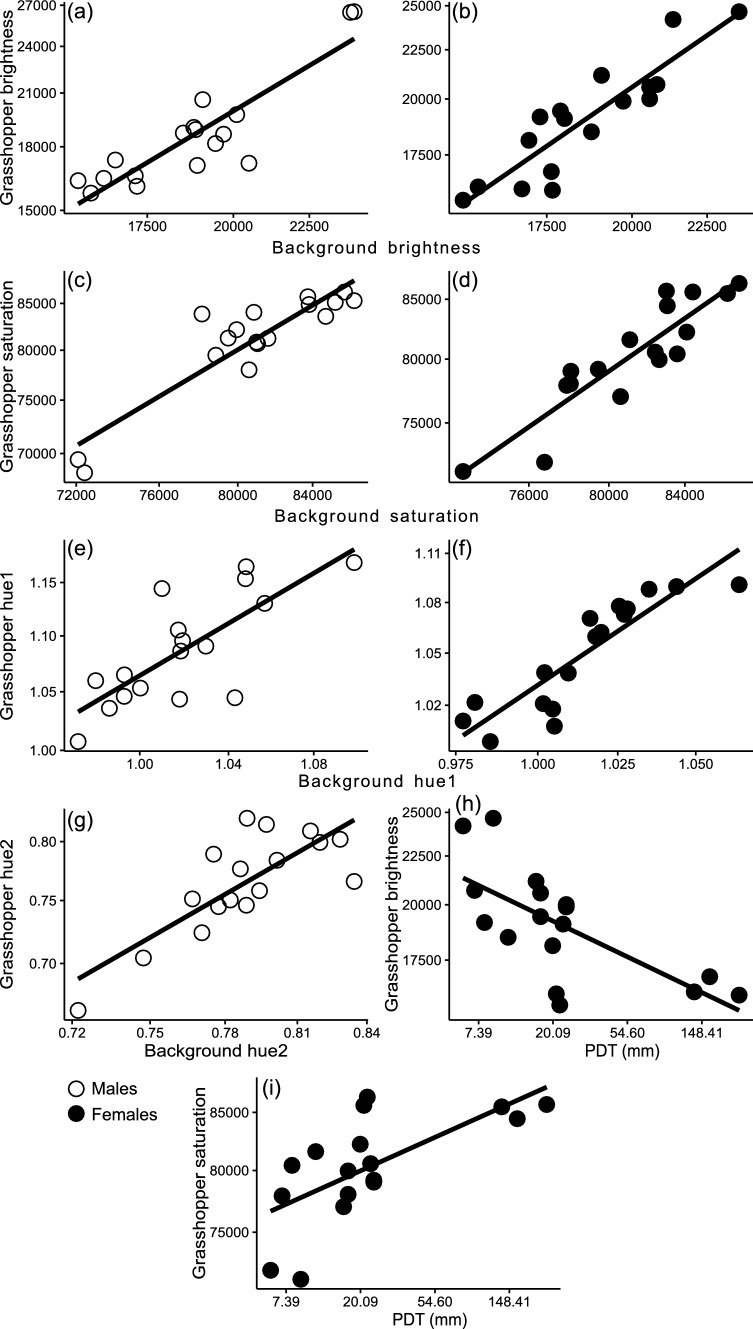
Associations between chromatic variables (brightness, saturation, hue1, hue2) of male (**a**, **c**, **e**, **g**) and female (**b**, **d**, **f**) *Sphenarium* grasshoppers and their backgrounds. Panels **h** and **i** illustrate the associations of female chromatic variables (brightness and saturation, respectively) with PDT (precipitation of the driest trimester). Ordinary least squares regressions are shown for illustrative purposes. Natural logarithmic scale used in all panels. The data were natural log-transformed prior to analyses. Open circles refer to male data; closed circles denote female data

The PGLS models in which we tested the association between coloration parameters show a general strong, positive association between the color parameters of male and female grasshoppers and their microhabitats (Table 1A–H; Fig. 2a–g). The only exception was hue2 in females (Table 1D). Moreover, the analyses show a significant positive association between the brightness and saturation of the females with the precipitation of the driest trimester of the year (Table 1A, B; Fig. 2h, i), males have a weak association that turned non-significant after the false rate discovery adjustment in brightness with the PWT (Table 1E). The values of λ are overall very low (zero or close to zero) (Table 1A–H), which suggests a lack of phylogenetic signal and a rapid evolution of these coloration traits.

**Table 1:** PGLS models of *Sphenarium* grasshoppers on coloration and pattern parameters for both sexes

Response variable	Explanatory variable	*T*	*F*	Multiple R^2^	P	Adj P	λ^**††**^
A) ♀ Brightness	♀ Bg-Brightness^†^	6.871	13.637		< 0.0001	**0.0002**	
	PDT^‡^	−3.131	2.831	0.861	0.008	**0.038**	0
	PWT^§^	0.929	0.249		0.369	0.533	
B) ♀ Saturation	♀ Bg-Saturation	7.253	15.200		< 0.0001	**0.0002**	
	PDT	3.119	2.811	0.867	0.008	**0.038**	0
	PWT	−1.320	0.503		0.209	0.400	
C) ♀ Hue 1	♀ Bg-Hue1	6.653	12.787		< 0.0001	**0.0002**	
	PDT	−0.849	0.208	0.770	0.411	0.533	0
	PWT	1.110	0.356		0.286	0.482	
D) ♀ Hue 2	♀ Bg-Hue2	1.866	1.005		0.084	0.222	
	PDT	1.497	0.648	0.335	0.158	0.339	0
	PWT	−0.853	0.210		0.409	0.533	
E) ♂ Brightness	♂ Bg-Brightness	6.013	10.445		< 0.0001	**0.0003**	
	PDT	−1.489	0.641	0.824	0.160	0.400	0.267
	PWT	2.437	1.716		0.030	0.105	
F) ♂ Saturation	♂ Bg-Saturation	6.558	12.423		< 0.0001	**0.0002**	
	PDT	1.142	0.377	0.833	0.274	0.480	0.214
	PWT	−2.117	1.295		0.054	0.175	
G) ♂ Hue 1	♂ Bg-Hue1	3.658	3.866		0.006	**0.017**	
	PDT	−0.081	0.002	0.514	0.936	0.936	0
	PWT	0.936	0.253		0.419	0.533	
H) ♂ Hue 2	♂ Bg-Hue2	4.957	3.005		0.0003	**0.002**	
	PDT	−0.71	0.148	0.702	0.490	0.605	0
	PWT	−1.998	0.121		0.067	0.201	
I) ♀ Overall patterns' contrast	♀ Bg-overall patterns’ contrast	1.483	0.635		0.161	0.334	
	PDT	−1.930	1.076	0.2702	0.076	0.212	0
	PWT	0.610	0.107		0.552	0.644	
J) ♀ Dominant marking size	♀ Bg-dominant marking size	0.552	0.088		0.872	0.893	
	PDT	0.167	0.008	0.1073	0.870	0.893	0
	PWT	−1.036	0.310		0.319	0.488	
K) ♀ Pattern diversity	♀ Bg-pattern diversity	2.901	2.431		0.012	0.052	
	PDT	−1.022	0.301	0.2630	0.325	0.488	0.603
	PWT	1.629	0.767		0.127	0.314	
L) ♂ Overall patterns' contrast	♂ Bg-overall patterns’ contrast	0.846	0.207		0.412	0.533	
	PDT	−2.444	1.725	0.315	0.029	0.104	0.588
	PWT	1.052	0.320		0.312	0.488	
M) ♂ Dominant marking size	♂ Bg-dominant marking size	0.439	0.056		0.667	0.737	
	PDT	0.618	0.110	0.335	0.547	0.644	0
	PWT	−1.364	0.538		0.196	0.391	
N) ♂ Pattern diversity	♂ Bg-dominant marking contrast	0.329	0.031		0.748	0.805	
	PDT	−1.147	0.380	0.335	0.272	0.479	0
	PWT	0.556	0.089		0.588	0.667	

All models Degrees of Freedom: 3, 13

^†^Bg: background

^‡^PDT: precipitation of the driest trimester of the year

^§^PWT precipitation of the wettest trimester

^**††**^ λ: Pagel’s Lambda. Prior to analyses data were natural log transformed

*Adj* P values after correction for false discovery rate

### *Association between grasshoppers' pattern and background pattern* + *climatic factors*

None of the PGLS of patterns was statistically significant after the false rate discovery test (Table 1I–L). However, the λ values were high for both female pattern diversity and male overall pattern’s contrast (Table 1K, L), which suggests high phylogenetic signal and slow evolution of traits.

### Sexual dimorphism in color patterns

**Fig. 3:**
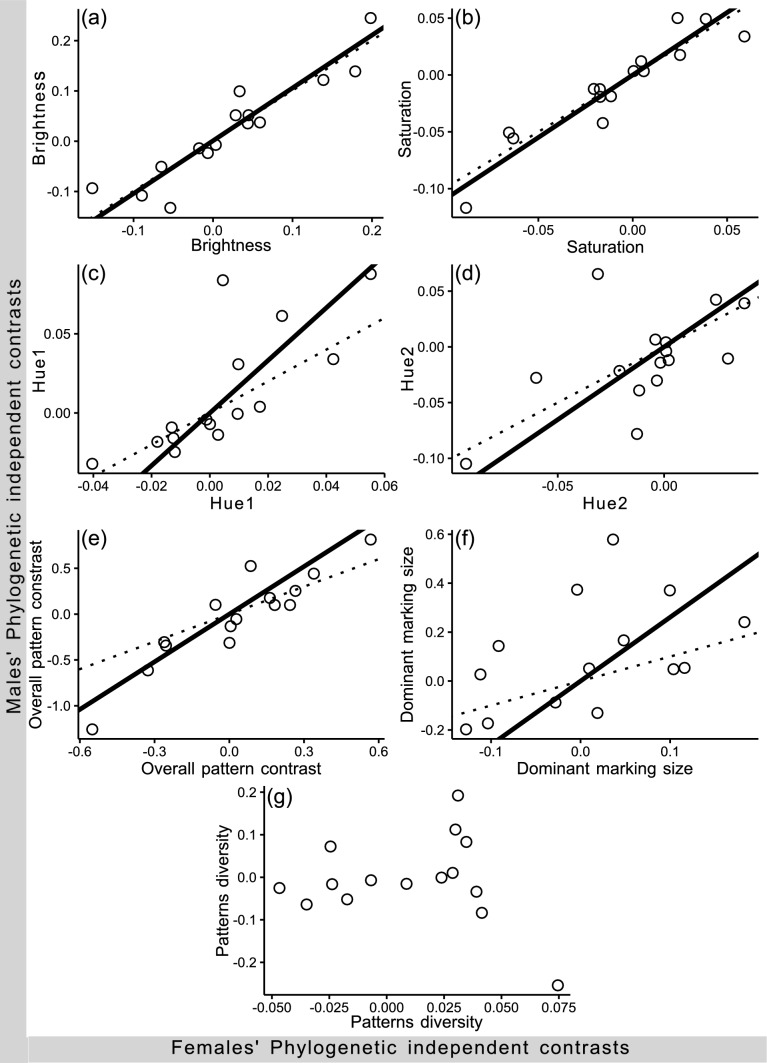
Major axis regressions of the phylogenetic independent contrasts (Solid lines) of the chromatic components of males on the chromatic components of the females of *Sphenarium* grasshoppers: **a** brightness, **b** saturation, **c** hue 1, **d** hue 2, **e** overall contrast patterns, **f** dominant marking size and **g** Pattern diversity. Dotted lines: Slope = 1

The independent contrasts of the major axis regressions of males on females for hue1 and 2, saturation, and brightness, overall patterns’ contrast and dominant marking size were positive and highly correlated, whereas the patterns diversity evolves independently in males and females (Table 2). The rate of divergence of brightness, saturation and hue2 was similar in males and females, while the divergence rate of hue1, overall patterns’ contrast and dominant marking size was higher on males than females (slope steeper than 1; Fig. 3 c, e, f).

**Table 2:** Major axis regressions using phylogenetic independent contrasts of chromatic patterns of *Sphenarium* males on the phylogenetic independent contrasts chromatic patterns of the females

Coloration /patterns traits	β^†^	UCL^‡^	LCL^§^	r^2^	r_w_^¶^	D.f	P
Brightness	1.05	1.29	0.85	0.86	0.13	13	0.6298
Saturation	1.10	1.34	0.9	0.87	0.26	13	0.3449
Hue1	1.66	2.37	1.16	0.59	0.63	13	**0.0096**
Hue2	1.30	2.02	0.84	0.37	0.32	13	0.2373
Overall patterns’ contrast	1.73	2.21	1.35	0.81	0.80	13	**0.0002**
Dominant marking Size	2.61	4.33	1.57	0.21	0.78	12	**0.0007**
Patterns’ diversity	− 20.4	–	–	0.002	–	–	–

^†^β: Coefficients of regressions

^‡^UCL and ^§^LCL: upper and lower 95% confidence intervals,

r_w_^¶^: Wald statistic values

## Discussion

We found a general positive association between the color parameters of male and female grasshoppers and their microhabitats. Additionally, the brightness and saturation of females were positively correlated with the precipitation during the driest trimester of the year. In contrast, males showed a weaker association and lacked phylogenetic signal between brightness and PWT, suggesting rapid evolution of this coloration trait. However, females exhibited a high phylogenetic signal for pattern diversity, while males displayed a strong signal for overall pattern contrast, indicating slow evolution of these traits. Furthermore, the rates of divergence between males and females for hue 1 and 2, saturation, brightness, overall pattern contrast, and dominant marking size were similar. Notably, males had higher divergence rates than females for hue 1, overall pattern contrast, and dominant marking size.

Our results suggest that the observed disparities in color patterns among the *Sphenarium* taxa can be attributed to localized adaptations to distinct environmental conditions. Moreover, a nuanced sex-specific divergence is discernible in their responses to environmental cues. Male coloration was associated to its surroundings in all chromatic patterns including hue2; the situation was the opposite in females, even though both sexes did not diverge in hue2. An interesting aspect that emerges is the relatively rapid evolution of chromatic patterns compared to the evolution of marking patterns. Perhaps, the interaction of some genes with environmental parameters such as temperature, humidity and food quality may explain the faster adaptive divergence of the chromatic patterns in *Sphenarium* grasshoppers (Dearn 1990; Tanaka 2004; Tanaka et al. 2012; Valverde and Schielzeth 2015).

The disparity in the pace of evolutionary divergence between background resemblance and markings can be explained by the differing advantages of each strategy. In scenarios where a species colonizes a new, vibrant, and uniform environment, natural selection often accelerates the convergence of coloration with the new surroundings, a strategy known as BM. On the other hand, if the pattern markings disrupt a predator's attention, they can be effective regardless of the background. Recent studies have demonstrated that disruptive markings significantly reduce predation in highly heterogeneous environments, even when there is no match in color or luminance between the prey and the substrate (Xiao and Cuthill 2016; Rowe et al. 2021). The possibly disruptive adaptability inherent in markings allows for a reduced divergence among species that face novel environments or alterations in their distribution range. In this context, due to the spatial and temporal chromatic heterogeneity, natural selection may favor a generalized strategy in which *Sphenarium* species moderately match various backgrounds, while not precisely mimicking one (de Alcantara Viana et al. 2023). It is conceivable that these grasshoppers adopt a form of camouflage that offers partial resemblance to multiple backgrounds rather than a perfect match to a singular habitat. Alternatively, their camouflage may operate somewhat autonomously from BM strategies (as posited by Hughes et al. 2019). Nevertheless, the apparent convergence between *Sphenarium* chromatic patterns and their environments stems from a blend of differential predation dynamics and proactive habitat selection. This phenomenon parallels other grasshopper species (as demonstrated by Edelaar et al. 2017, 2019; Heinze et al. 2022).

The survival of these grasshoppers hinges on their ability to navigate diverse predator threats, including avian, mammalian, reptilian, and arthropod predators (Kevan 1977), as well as human predation (highlighted by Sanabria-Urbán and Cueva del Castillo 2020). Their chromatic and achromatic visual cues have evolved in response to the distinct searching strategies of these predators. Remarkably, the resemblance of marking patterns to the environment extends to chromatic and achromatic variables. However, while chromatic cues excel in close-range searches, achromatic information proves more effective in long-distance prey detection (Schaefer and Stobbe 2006; Cazetta et al. 2009). Notably, human behavior as a predator mirror that of natural predators, particularly birds, a similarity exploited in some studies to predict predation by other visual predators in natural settings (Karpestam et al. 2013; de Alcantara Viana et al. 2022). This intriguing correspondence has likely influenced the evolution of *Sphenarium* grasshoppers' chromatic patterns.

It is worth noting that the benefits derived from cryptic strategies differ between males and females. The divergence rate of hue1, overall pattern contrast, and dominant marking size are notably higher in males than in females. Conversely, the evolution of pattern diversity has proceeded independently within both genders, indicating a distinct niche specialization concerning light and shadow contrast for males and females. Nonetheless, the parallel evolution rates of brightness and saturation in males and females can be attributed to similar selective pressures faced by different species within their respective habitats. Interestingly, hue2 evolved at the same rate as brightness and saturation but was only linked to the environment in males. This suggests that males and females either utilize their respective habitats differently or exhibit distinct behaviors that lead them to occupy niches with varying chromatic compositions, with males likely blending more effectively (Cueva del Castillo et al. 2021). In *S. purpurascens*, females tend to be less mobile than males and are often found near the ground, where they lay their eggs. In contrast, males are typically observed on top of plants, actively searching for females (R. Cueva del Castillo pers. obs.).

Considering the relative scarcity of phylogenetic comparative studies concerning cryptic coloration evolution, our research undoubtedly contributes to a foundational comprehension of color evolution within the context of selection pressures imposed by visual predators. Nonetheless, it is imperative to acknowledge that future investigations into habitat preference and the exploration of other potential functions of grasshopper coloration, such as thermoregulation or UV radiation protection, hold the potential to unveil additional factors shaping the evolution of coloration within this cohort of neotropical grasshoppers.

## Supplementary Information

Below is the link to the electronic supplementary material.

Supplementary file1 (DOCX 470 KB)

## Data Availability

Data will be available under reasonable request.
