# Supplementary material for: A macroevolutionary perspective of cryptic coloration in sexually dichromatic grasshoppers of the genus Sphenarium (Orthoptera: Pyrgomorphidae)
Source: Oecologia. 2025 Jan 10;207(1):19. doi: 10.1007/s00442-024-05643-7 (PMC11723891; doi:10.1007/s00442-024-05643-7)
Supplement: Supplementary file 1 — Supplementary file1 (DOCX 470 KB) [file 442_2024_5643_MOESM1_ESM.docx]

**Supplementary Material**

**Fig. S1.** Map of the sites where the photographs of *Sphenarium* grasshoppers were taken: *S. purpurascens* (19.31N, 99.19W), *S. borrei* (19.92N, 101.74W), *S. occidentalis* (18.66N, 101.65W), *S. cypticum* (18.18N, 100.65W), *S.rugosum* (18.68N, 99.53W), *S. planum* (18.48N, 97.45W), *S. histrio* (16.93N, 96.42N), *S. variabile* (16.93N, 96.42N), *S. miztecum* (16.37N, 98.47W), *S. adelinae* (17.43N, 99.47W), *S. macrophallicum* (19.38N, 100.39W), *S. tarascum* (19.20N, 101.73W), *S. infernalis* (18.94N, 101.81W), *S. zapotecum* (17.07N, 97.83N), *S. mexicanum* (17.75N, 96.31W), *S. minimum* (19.09N, 97.03W), and *S. totonacum* (19.72N, 96.68W)


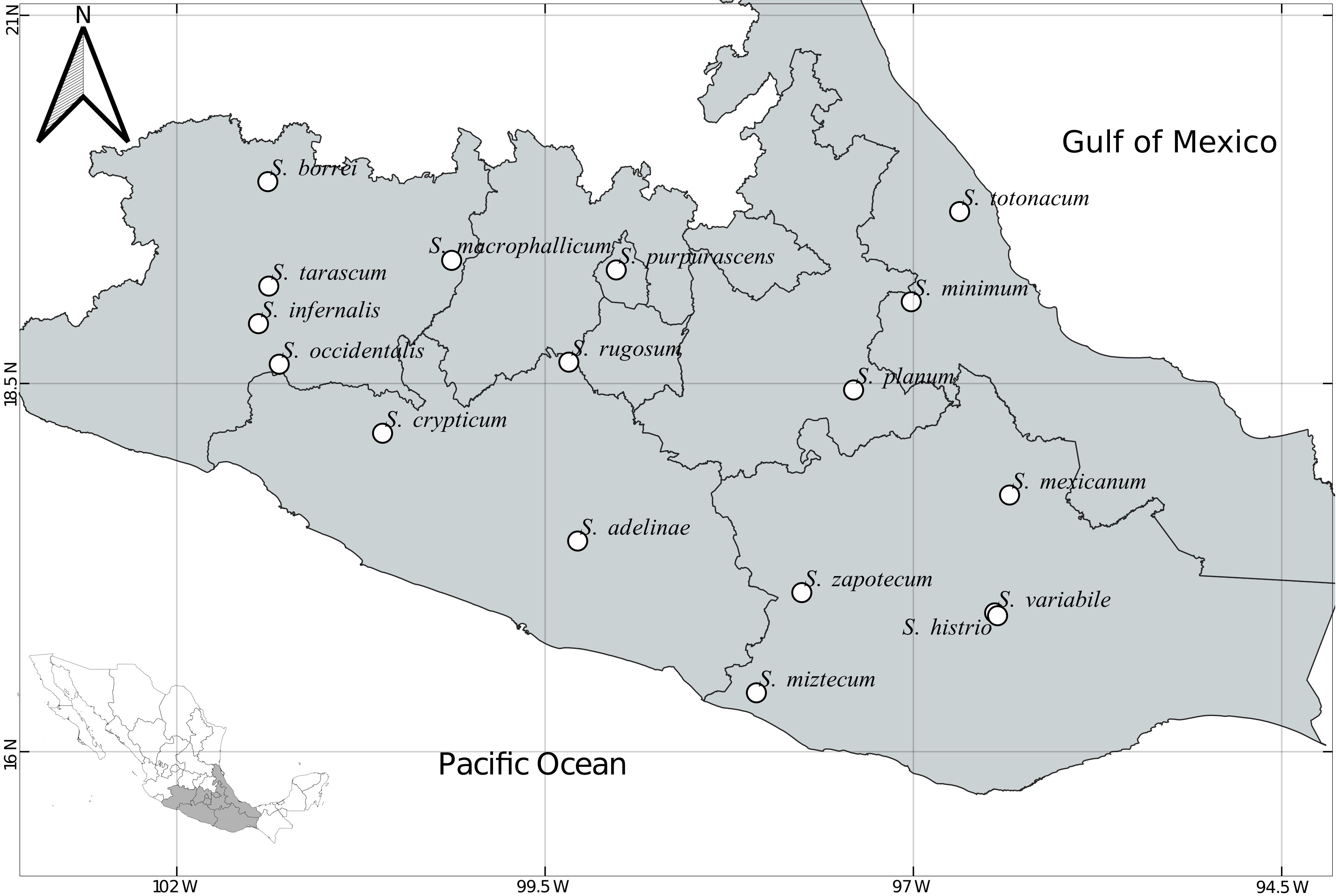


| Table S1. Sites where *Sphenarium* species were collected. Locality names, geographic coordinates, and their elevation. | | | |
| --- | --- | --- | --- |
| Species | Locality name | Geographic Coordinates | Elevation (masl) |
| *S. purpurascens* | Pedregal de San Angel, Ciudad de México | 19.31N, 99.19W | 2324 |
| *S. borrei* | Villa Jiménez, Michoacán | 19.92N, 101.74W | 1985 |
| *S. occidentalis* | Churumuco, Michoacán | 18.66N, 101.65W | 190 |
| *S. cypticum* | Near Ciudad Altamirano, Michoacán | 18.18N, 100.65W | 540 |
| *S. rugosum* | Near Cacahuamilpa, Guerrero | 18.68N, 99.53W | 1523 |
| *S. planum* | Tehuacán, Puebla | 18.48N, 97.45W | 1731 |
| *S. histrio* | Near Mitla, Oaxaca | 16.93N, 96.42W | 1653 |
| *S. variabile* | Near Mitla, Oaxaca | 16.93N, 96.42W | 1653 |
| *S. miztecum* | Near Santiago Tepextla, Oaxaca | 16.37N, 98.47W | 77 |
| S. adelinae | Near Chilpancingo, Guerrero | 17.43N, 99.47W | 1298 |
| *S. macrophallicum* | Presa del Bosque, Michoacán | 19.38N, 100.39W | 1760 |
| *S. tarascum* | Ario de Rosales, Michoacán | 19.20N, 101.73W | 1939 |
| S. infernalis | Near La Huacana, Michoacán | 18.94N, 101.81W | 575 |
| S. zapotecum | San Isidro Paz y Progreso, Oaxaca | 17.07N, 97.83W | 1563 |
| *S. mexicanum* | Yetla, Oaxaca | 17.75N, 96.31W | 202 |
| *S. minimum* | Near Cosomatepec de Bravo, Veracruz | 19.09N, 97.03W | 1479 |
| *S. totonacum* | Tierra Blanca, Veracruz | 19.72N, 96.68W | 1090 |

| Table S2. Chromatic and precipitation data for females and males of 17 grasshopper species of the genus *Sphenarium. M: Male; F: Female. n*: Sampled size; Grasshoppers Hue1 and Hue 2: G Hue1, G Hue 2; Background Brightness: B Brightness; Background Saturation: B Saturation; Background Hue1 and Hue 2: B Hue1, B Hue2; Grasshoppers overall patterns contrast: G OPS; Grasshoppers dominant marking size: G DMS; Grasshopper patterns diversity: G PD; Background overall patterns contrast: B OPC; Background dominant marking size: B DMS; Background patterns diversity: B PD; Precipitation of the driest trimester (mm):PTD; Precipitation of the wettest trimester (mm): PWT. | | | | | | | | | | | | | | | | | | |
| --- | --- | --- | --- | --- | --- | --- | --- | --- | --- | --- | --- | --- | --- | --- | --- | --- | --- | --- |
| Species | Sex | *n* | Brightness | Saturation | G Hue1 | G Hue2 | B Brightness | B Saturation | B Hue1 | B Hue2 | G OPC | G DMS | G PD | B OPC | B DMS | B PD | PDT | PWT |
| *S. adelinae* | F | 18 | 18120.06 | 82222.30 | 1.04 | 0.78 | 17018.47 | 84098.96 | 1.01 | 0.81 | 419.41 | 36.92 | 0.10 | 234.67 | 22.20 | 0.11 | 20 | 690 |
| *S. adelinae* | M | 22 | 20603.26 | 78002.31 | 1.14 | 0.75 | 19071.46 | 80588.84 | 1.01 | 0.79 | 1656.26 | 20.73 | 0.13 | 363.22 | 35.60 | 0.11 | 20 | 690 |
| *S. borrei* | F | 20 | 19090.26 | 80570.76 | 1.07 | 0.76 | 17992.41 | 82418.64 | 1.02 | 0.81 | 502.54 | 40.94 | 0.11 | 403.98 | 45.15 | 0.11 | 23 | 595 |
| *S. borrei* | M | 10 | 18167.83 | 82160.64 | 1.09 | 0.75 | 19453.90 | 79937.14 | 1.02 | 0.78 | 573.91 | 25.71 | 0.11 | 489.10 | 21.99 | 0.12 | 23 | 595 |
| *S. crypticum* | F | 5 | 19162.99 | 80427.77 | 1.02 | 0.76 | 17317.53 | 83586.36 | 0.98 | 0.80 | 539.56 | 38.47 | 0.10 | 223.74 | 18.59 | 0.10 | 8 | 753 |
| *S. crypticum* | M | 17 | 17330.72 | 83585.20 | 1.04 | 0.76 | 16656.08 | 84723.73 | 0.99 | 0.79 | 552.10 | 13.96 | 0.11 | 172.98 | 12.70 | 0.10 | 8 | 753 |
| *S. histrio* | F | 27 | 19436.35 | 79959.28 | 1.06 | 0.77 | 17879.71 | 82626.50 | 1.02 | 0.80 | 542.42 | 43.66 | 0.10 | 317.32 | 32.10 | 0.11 | 17 | 305 |
| *S. histrio* | M | 22 | 18907.22 | 80845.44 | 1.11 | 0.82 | 18861.69 | 80983.23 | 1.02 | 0.79 | 1161.15 | 13.39 | 0.13 | 365.14 | 32.58 | 0.11 | 17 | 305 |
| *S. infernalis* | F | 8 | 20707.17 | 77904.95 | 1.01 | 0.77 | 20799.73 | 77866.38 | 1.01 | 0.75 | 582.94 | 56.14 | 0.12 | 524.26 | 55.19 | 0.11 | 7 | 562 |
| *S. infernalis* | M | 12 | 19738.88 | 79488.58 | 1.04 | 0.75 | 20103.50 | 78860.48 | 1.04 | 0.77 | 747.60 | 31.60 | 0.11 | 432.85 | 49.56 | 0.12 | 7 | 562 |
| *S. macrophallicum* | F | 17 | 20011.63 | 79028.64 | 1.09 | 0.76 | 20562.32 | 78076.89 | 1.04 | 0.77 | 495.93 | 44.77 | 0.11 | 437.96 | 31.94 | 0.12 | 24 | 550 |
| *S. macrophallicum* | M | 15 | 18724.45 | 81234.70 | 1.09 | 0.72 | 18505.84 | 81591.29 | 1.03 | 0.77 | 644.76 | 24.54 | 0.10 | 405.44 | 21.14 | 0.11 | 24 | 550 |
| *S. mexicanum* | F | 13 | 16082.06 | 85708.00 | 1.00 | 0.80 | 17657.35 | 83010.61 | 0.99 | 0.82 | 297.34 | 39.11 | 0.10 | 253.40 | 45.46 | 0.10 | 245 | 2065 |
| *S. mexicanum* | M | 16 | 16335.67 | 85300.20 | 1.01 | 0.77 | 15722.06 | 86310.18 | 0.97 | 0.83 | 490.50 | 12.72 | 0.11 | 143.29 | 16.26 | 0.10 | 245 | 2065 |
| *S. minimum* | F | 15 | 16209.63 | 85498.14 | 1.01 | 0.77 | 15715.47 | 86323.82 | 0.98 | 0.83 | 235.56 | 36.60 | 0.12 | 126.46 | 28.20 | 0.11 | 134 | 1022 |
| *S. minimum* | M | 12 | 15762.27 | 86258.80 | 1.06 | 0.80 | 16036.23 | 85768.66 | 0.98 | 0.83 | 264.45 | 22.14 | 0.12 | 179.12 | 28.03 | 0.11 | 134 | 1022 |
| *S. miztecum* | F | 24 | 24192.45 | 72072.83 | 1.08 | 0.68 | 21331.20 | 76769.07 | 1.03 | 0.78 | 728.95 | 29.77 | 0.11 | 487.96 | 25.71 | 0.11 | 6 | 951 |
| *S. miztecum* | M | 28 | 26505.17 | 69450.37 | 1.15 | 0.70 | 24119.28 | 72086.76 | 1.05 | 0.75 | 2809.16 | 14.47 | 0.14 | 650.25 | 20.71 | 0.11 | 6 | 951 |
| *S. occidentalis* | F | 17 | 24645.27 | 71399.37 | 1.07 | 0.67 | 23656.96 | 72862.32 | 1.03 | 0.73 | 709.06 | 34.26 | 0.11 | 327.79 | 74.06 | 0.11 | 9 | 398 |
| *S. occidentalis* | M | 10 | 26454.81 | 68280.15 | 1.17 | 0.66 | 23984.26 | 72390.42 | 1.10 | 0.72 | 1581.81 | 13.66 | 0.14 | 670.16 | 17.09 | 0.13 | 9 | 398 |
| *S. planum* | F | 43 | 18490.40 | 81589.16 | 1.02 | 0.76 | 18768.93 | 81085.47 | 1.00 | 0.80 | 263.54 | 43.45 | 0.10 | 431.07 | 41.30 | 0.11 | 11 | 234 |
| *S. planum* | M | 34 | 17064.07 | 84025.50 | 1.04 | 0.78 | 18910.83 | 80844.94 | 1.02 | 0.80 | 306.49 | 61.31 | 0.11 | 449.75 | 18.80 | 0.11 | 11 | 234 |
| *S. purpurascens* | F | 43 | 19896.90 | 79189.40 | 1.08 | 0.77 | 19726.22 | 79459.15 | 1.03 | 0.79 | 433.46 | 31.56 | 0.11 | 780.92 | 37.59 | 0.11 | 24 | 501 |
| *S. purpurascens* | M | 44 | 17172.25 | 83843.16 | 1.05 | 0.78 | 20488.75 | 78152.29 | 1.00 | 0.79 | 496.64 | 28.00 | 0.11 | 966.32 | 14.77 | 0.12 | 24 | 501 |
| *S. rugosum* | F | 26 | 16132.21 | 85629.48 | 1.04 | 0.79 | 16833.31 | 84425.40 | 1.00 | 0.79 | 376.69 | 43.16 | 0.11 | 186.74 | 20.26 | 0.10 | 21 | 676 |
| *S. rugosum* | M | 35 | 16073.29 | 85730.63 | 1.06 | 0.81 | 17225.75 | 83739.06 | 0.99 | 0.82 | 376.69 | 43.16 | 0.11 | 213.88 | 23.16 | 0.10 | 21 | 676 |
| *S. tarascum* | F | 18 | 21166.79 | 77024.98 | 1.06 | 0.76 | 19067.58 | 80604.10 | 1.02 | 0.79 | 593.72 | 50.83 | 0.11 | 301.94 | 36.77 | 0.11 | 16 | 762 |
| *S. tarascum* | M | 33 | 19028.84 | 80687.47 | 1.10 | 0.75 | 18809.87 | 81035.64 | 1.02 | 0.78 | 751.02 | 24.46 | 0.12 | 354.27 | 36.12 | 0.11 | 16 | 762 |
| *S. totonacum* | F | 8 | 16813.65 | 84444.38 | 1.02 | 0.79 | 17627.16 | 83056.94 | 1.00 | 0.78 | 519.50 | 37.66 | 0.11 | 323.19 | 61.56 | 0.12 | 165 | 713 |
| *S. totonacum* | M | 9 | 16564.54 | 84877.03 | 1.05 | 0.80 | 17177.45 | 83808.83 | 0.99 | 0.82 | 523.30 | 30.65 | 0.11 | 227.19 | 52.01 | 0.11 | 165 | 713 |
| *S. variabile* | F | 38 | 20573.09 | 78026.30 | 1.09 | 0.77 | 20561.92 | 78054.94 | 1.06 | 0.79 | 610.56 | 46.30 | 0.11 | 349.01 | 25.56 | 0.11 | 17 | 305 |
| *S. variabile* | M | 22 | 18660.26 | 81268.61 | 1.13 | 0.82 | 19697.72 | 79499.34 | 1.06 | 0.80 | 1086.42 | 14.25 | 0.13 | 422.10 | 27.67 | 0.11 | 17 | 305 |
| *S. zapotecum* | F | 18 | 15702.49 | 86373.98 | 1.09 | 0.79 | 15349.90 | 86971.74 | 1.03 | 0.80 | 317.52 | 28.17 | 0.11 | 177.96 | 32.36 | 0.11 | 22 | 893 |
| *S. zapotecum* | M | 33 | 16443.98 | 85109.07 | 1.17 | 0.79 | 16359.43 | 85250.42 | 1.05 | 0.78 | 852.79 | 12.69 | 0.14 | 253.45 | 33.35 | 0.11 | 22 | 893 |
